# Developmental Epigenetics: Phenotype and the Flexible Epigenome

**DOI:** 10.3389/fcell.2018.00130

**Published:** 2018-10-11

**Authors:** Rosalind M. John, Claire Rougeulle

**Affiliations:** ^1^Biomedicine Division, Cardiff School of Biosciences, Cardiff University, Cardiff, United Kingdom; ^2^Sorbonne Paris Cité, Epigenetics and Cell Fate, UMR 7216 CNRS, Université Paris Diderot, Paris, France

**Keywords:** epigenetics, development, adaptation, evolution, phenotype

The term “epigenetics” has been widely used and abused (Greally, 2018) but the most compelling definition of epigenetics is the study of changes in gene function that are heritable through cell division, yet reversible, and that do not involve changes in DNA sequence - with heritability and reversibility being the key factors. Epigenetic information persists after the original inductive process that drove the modification has ceased, providing a cellular memory of the process or exposure in subsequent generations. Epigenetic marks allow the cell to remember what kind of cell it is irrespective of positional information and other extracellular information. Parent cells use epigenetic marks to “tell” their daughter cells what type of cell they will become, a message that may persist through thousands of cell divisions for the lifetime of the organism, unless they are actively erased or lost through epimutation. Epigenetic processes are fundamentally important for cell identity, lineage determination, regeneration and re-establishing of the next generation. They explain how an identical set of genomic instructions can generate all the required cell types for the organism without the need, in most cases, to alter gene sequence.

The heritability through mitosis of the epigenetic information is relatively well characterized and acknowledged by the scientific community. Studies in various organisms, including plants and nematodes, have also revealed that epigenetic traits can be propagated through meiosis i.e., from one generation to the next. There is, however, much debate as to whether this holds true in mammals. The reason behind this questioning is the extensive epigenetic reprogramming that occurs twice in mammalian life, namely during the formation of the gametes and, after their fusion, in the embryo to be implanted. These events lead to an *a priori* complete (and this is where part of the debate stands) erasure of the epigenetic information that has been acquired during the parent's life, so that the new generation starts with a “clean slate.” In addition, it is difficult to confidently assign the heritability of a given molecular character that is acquired following exposure to stress or stimuli, solely to epigenetic information rather than a subtle and perhaps hard to track change in the underlying genetic material. From a molecular view, classic epigenetic marks include DNA methylation and the modification of proteins that lie on or over the DNA sequence itself (Cedar and Bergman, 2009). Chromatin and epigenetic are not, however, interchangeable terms. Chromatin-based mechanisms of gene regulation are not necessarily epigenetic, at least not more than “classical” regulatory processes involving transcription factors. It is, again, a matter of heritability of a status in the absence of the original trigger. Epigenetics can also involve non-coding RNA molecules, small and long, providing they are passed from one cell to another or from one generation to the next to maintain phenotype (Chen et al., 2016). There is, for example, limited argument to consider microRNAs, which control mRNA (and other types of RNA) stability and translation, as epigenetic regulators. Developmental epigenetics is not the study of these inherited factors *per se*, nor their global distribution across the genome, but is the study of the function of epigenetic processes during development, studies which may include the developmental programming of fetal growth trajectories and adult phenotypes.

## Functional epigenetics

A key challenge in the discipline is directly linking changes in epigenetic marks with phenotypic outcomes. While there are numerous published studies on epigenetic differences between states, findings are correlative, and causality is not well established (Greally, 2017). We can show that specific epigenetic modifications affect the accessibility of genomic regions such as promoters, preventing or allowing transcription factors or protein complexes to bind. This in turn can alter local chromatin structure or direct transcription. It is also possible to demonstrate experimentally that substantial changes in DNA methylation result in significant changes in gene transcription, fundamentally acting as on/off switches. Deletion of epigenetic regulators such as the DNA methyltransferases has profound consequences for gene transcription and development (Lyko, 2018). Epigenetic marks are also demonstrably critical for the formation and maintenance of heterochromatin (Saksouk et al., 2015), and for developmental processes such as X-chromosome inactivation (Brockdorff, 2017) and genomic imprinting (John and Surani, 1996). However, in each example multiple layers of epigenetic marks function as aggregates to control transcription. To what extent do small changes, even changes at single CpG sites or individual histone tails, have significant functional consequences that can be maintained and propagated upon division? A further question is the developmental relevance of epigenetic modifications that lie outside promoter or enhancer regions. Technologies such as next-generation sequencing (NGS) and single cell analysis enable us to quantify subtle epigenetic differences in great detail. However, new approaches are needed to alter single epigenetic modifications or subtly modify groups of marks in order to test their functional relevance. In this respect, epi-editing holds great promise (Liu et al., 2016; Thakore et al., 2016). Epigenetic modulators can be fused to catalytically inactive Cas9 (dCas9) or TALENs to enable targeted DNA methylation or histone modification, editing events that can drive the activation or silencing of a target locus or gene and, importantly, test the functional relevance of epigenetic marks over time. These strategies will be instrumental in addressing the consequences of transmitting epigenetic information across generations.

## Fetal programming

A related challenge within the field of developmental epigenetics is understanding the link between early life exposures and later life outcomes. Human epidemiological studies have repeatedly linked adverse intrauterine and early postnatal events with subsequent obesity and metabolic disease as well as higher risk of a number of common mental health conditions—a phenomenon termed “fetal programming” (Barker, 1990) or developmental origins of disease (Gluckman and Hanson, 2004). During development the epigenome undergoes extensive modification with epigenetic remodeling driving cellular differentiation to establish cell- and tissue-specific pattern of gene expression. Concurrently, in mammals the germline is undergoing waves of erasure and reestablishment of epigenetic marks to reprogram the epigenome for the next generation (Perino and Veenstra, 2016; Iurlaro et al., 2017; Okada and Yamaguchi, 2017). Epigenetic dysregulation had been proposed as a potential mechanism underlying anomalous fetal programming. It certainly fits the bill as epigenetic marks, unlike DNA sequence, are flexible and can be both added and removed within the cell cycle. Critically, epigenetic marks can be “remembered” for the full lifetime of the individual.

In humans, numerous prenatal risk factors have been linked to poor health later in life including suboptimal maternal nutrition and maternal stress (Wu et al., 2004; Janssen et al., 2016). Early postnatal exposures such as the quality of maternal care can also influence later life outcomes, both negatively and positively (Curley and Champagne, 2016). Exposures rarely occur in isolation and there are different patterns and long-term consequences of fetal adversities depending on the timing, nature and extent of the insult, as well as the gender of the exposed individual, adding an additional layer of complexity. In spite of species related differences, animal studies are important in clarifying causality, and exploring resilience, reversibility, temporal, tissue and gender specific sensitivities to different exposures. Even with everything we have learned about epigenetic processes, and despite hundreds of intervention studies, we still do not know definitively that epigenetic mechanisms are responsible for fetal programming. Critically, seemingly different exposures can have the same phenotypic outcome while the same exposure at a different time point or for a different duration can have a significantly different phenotypic outcome. One possible explanation is that specific and discrete regions of the developing epigenome are exquisitely sensitive to insults *per se*, and that windows of vulnerability vary with the developmental time point, between different tissues and between males and females. Excellent candidates for these sensitive regions are the imprinted genes. Imprinted genes are expressed predominantly from one parental allele as a consequence of epigenetic events initiated in the germline and built on in somatic cells to generate domains of allele-specific epigenetic modification and gene expression, some of which span many megabases (Andergassen et al., 2017). Imprinted genes regulate fetal growth, placental development, postnatal metabolism and numerous complex mammalian behaviors (Cleaton et al., 2014). Imprinted genes may not necessarily be more responsive to prenatal insults but small changes in their expression can have significant phenotypic consequences that persist into adulthood and imprinted genes expressed in one individual can even impact the behavior of another individual (Creeth et al., 2018; McNamara et al., 2018). Poor diet in pregnancy is already known to alter the epigenetic regulation of at least some of these remarkable genes (Van de Pette et al., 2017).

An interesting and related question is whether prenatal insults impact the activity of the X chromosomes in females, one of which in epigenetically inactivated. X-inactivation is set up early *in utero* and controls the expression dosage for most of the ~1000 genes that mammalian X chromosomes carry (Sahakyan et al., 2017). While female development cannot be pursued in the absence of X-inactivation, more subtle dosage aberrancy of particular X-linked genes may, as for imprinted genes, have long term phenotypic consequences, in a gender-specific manner. Comprehensive screens of the full range of early life challenges in one model organism under fully controlled conditions are required to test these hypotheses properly. Given extraordinary developments in next generation bisulphite sequencing technology, it is now possible to look both at tissue-specific epigenetic/transcriptional signatures and the signatures of specific cell types within tissues, including the potentially most vulnerable stem cell populations. Developments in imaging technologies will also provide a new platform for these types of study increasing our capacity to detect subtle changes in gene expression (Van de Pette et al., 2017). Descriptions of epigenetic alterations alone, however, are not sufficient. Linking specific gene changes to phenotype is essential.

## Epigenetics and adaptation

**Figure 1:**
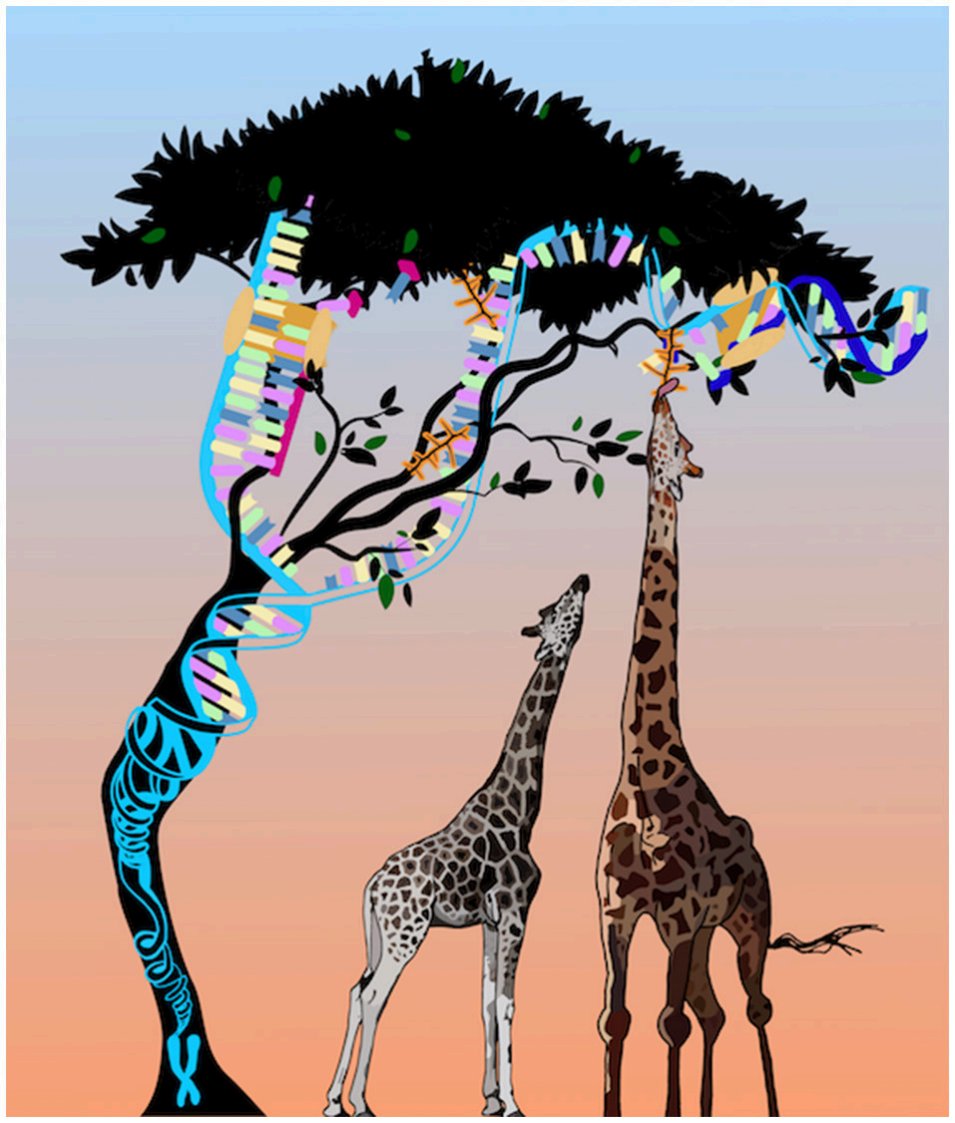
An update on Larmarck's giraffes.

A third challenge is understanding the role of epigenetic marks in environmental adaptations, and, as an extension of this concept—in evolution. Normally we view alterations in epigenetic marks as a negative outcome (epimutations), for example in case of certain cancers or the imprinting disorders. However, epigenetic flexibility may contribute to enhanced survival under different environmental conditions. There are examples of this in plants (Lämke and Bäurle, 2017) and some lower animals (Vogt, 2017) but again, the challenge is establishing cause and effect relationships. Unless reproduction is clonal in the wild, there are both genetic and epigenetic differences. Over 200 years ago Jean Baptiste Lamarck (1744-1829) proposed that environmental factors could lead to the increase or decrease of a particular structure and be passed on to offspring, giving the example of a giraffe stretching its neck to reach the juiciest leaves at the top of trees and then giving birth to progeny with similarly long necks (de Lamarck, 1830) (Figure 1). His theories contributed to the onset of Darwinism but were largely derided at the time. Now that we know epigenetic marks can respond to the environment and may not be fully erased in the germline, Lamarck's ideas are no longer quite so easily dismissed.

## Summary

In summary, a key area of focus for this specialty section on developmental epigenetics is understanding the functional relevance of both large and small changes in epigenetic marks in development and beyond. Connected with this work are studies investigating how early environmental exposures modulate epigenetic marks to alter later life phenotypes, with a critical emphasis on studies that establish causality. Finally, it is important to consider how epigenetic processes have contributed to evolution. Frontiers in Cell and Developmental Biology will serve as an important platform for studies in these areas and, like the epigenome, we will be flexible in response to our environmental cues (the epigenetics community) to take on emerging themes.

## Author contributions

Both authors listed have made a substantial, direct and intellectual contribution to the work, and approved it for publication.

### Conflict of interest statement

The authors declare that the research was conducted in the absence of any commercial or financial relationships that could be construed as a potential conflict of interest.
